# DNA Barcoding of Freshwater Fishes of Indo-Myanmar Biodiversity Hotspot

**DOI:** 10.1038/s41598-018-26976-3

**Published:** 2018-06-05

**Authors:** Anindya Sundar Barman, Mamta Singh, Soibam Khogen Singh, Himadri Saha, Yumlembam Jackie Singh, Martina Laishram, Pramod Kumar Pandey

**Affiliations:** College of Fisheries (Central Agricultural University, Imphal), Lembucherra, Tripura (West) 799210 India

## Abstract

To develop an effective conservation and management strategy, it is required to assess the biodiversity status of an ecosystem, especially when we deal with Indo-Myanmar biodiversity hotspot. Importance of this reaches to an entirely different level as the hotspot represents the area of high endemism which is under continuous threat. Therefore, the need of the present study was conceptualized, dealing with molecular assessment of the fish fauna of Indo-Myanmar region, which covers the Indian states namely, Manipur, Meghalaya, Mizoram, and Nagaland. A total of 363 specimens, representing 109 species were collected and barcoded from the different rivers and their tributaries of the region. The analyses performed in the present study, i.e. Kimura 2-Parameter genetic divergence, Neighbor-Joining, Automated Barcode Gap Discovery and Bayesian Poisson Tree Processes suggest that DNA barcoding is an efficient and reliable tool for species identification. Most of the species were clearly delineated. However, presence of intra-specific and inter-specific genetic distance overlap in few species, revealed the existence of putative cryptic species. A reliable DNA barcode reference library, established in our study provides an adequate knowledge base to the groups of non-taxonomists, researchers, biodiversity managers and policy makers in sketching effective conservation measures for this ecosystem.

## Introduction

Spread over an approximate expanse of two million square kilometers of tropical Asia, the Indo-Myanmar region is a kaleidoscope of an amazing geographical diversity and is among the top 10 biodiversity hotspots of the world. Stunning variation in terms of landforms and climatic zones supports an enormous variety of habitats, areas of endemism and plethora of biodiversity of the region. Ecology and lives of the people of the region are shaped by sweeping expanses of Asia’s mighty rivers such as Mekong, Chao Phraya, Irrawaddy, Salween, Chindwin, Sittaung, Red and Pearl (Information adapted from Ecosystem Profile of Indo-Burma Biodiversity Hotspot 2011 update, prepared by the Critical Ecosystem Partnership Fund, CEPF). Freshwater fishes support rural livelihoods in the region but they are among the most threatened organism in Indo-Myanmar region^1^, due to unsustainable fishing practices, invasive species, and habitat alteration and loss. Further, level of threat to the entire river ecosystem of the region could drastically increase in near future due to several anthropogenic activities. Therefore, sustainable management of the aquatic resources of the region is desperately needed to conserve the icthyofauna. A prerequisite for this is a careful and accurate assessment of fish species to devise appropriate conservation measures for the target species.

Paramount works have been done by several ichthyologists on exploration, but available data on the fish fauna of the region is incomplete and inconclusive. Reason for this includes a lack of extensive survey due to difficult hilly terrain, inaccessibility and prevalence of many indigenous languages/dialects for communication with various ethnic communities of the region. A total of 291 species has been reported from the entire northeast India as per the records of Zoological Survey of India^2^. Drainage-wise exploration studies are scanty in the region^3–5^. Majority of the studies on ichthyofauna of the region are limited to the description of new species and fish diversity, entirely based on morphometric & meristic characters and phylogenetics^2,6–14^. Further, species identification through morphometric and meristic characters often leads to misidentification, taxon ambiguities and fluctuation in species number. The main culprits for this include phenotypic and genotypic plasticity, cryptic diversity or possible hidden species and variation in color pattern at different stages of life history of the same species.

Pioneers of DNA barcoding proposed the method of molecular taxonomy or systematic, employing standardized and authenticated DNA based approach to provide accurate and automated species identification, using nucleotide sequence of partial region of mitochondrial gene, i.e. cytochrome oxidase I (COI)^15^. Like several other methods, DNA barcoding approach was also debated and divided by two schools of researchers, one questioned the claims made by barcoding^16–19^ and another supported the taxonomic resolution efficiency, accuracy and rapidity of the method^20–29^. Further, use of DNA barcoding is not restricted to species identification only. It also helps in flagging new species^5,25,30^, identification of raw fish material used in the value-added products^31–33^, identification of larvae and juveniles, collected from natural ecosystems for identification of breeding grounds and assessment of different life history stages^34,35^, identification of invasive alien species and forensics, including illegal wildlife harvesting^36–38^. In view of public interest, and in an effort to solve species substitution problem in the United States, US Food and Drug Administration (FDA) agency emphasized on the use of DNA barcoding of fishes for regulatory compliance in export, import and marketing of seafood^39,40^.

The Lingua Franca of traditional taxonomic methods with DNA barcode gap estimation not only strengthens the species identification, but also offers a new perspective to fish diversity assessment. Various automatic, fast and straightforward methods for performing species delimitation process, using specific bioinformatics analyses like Generalized Mixed Yule Coalescent (GMYC)^41–43^, Bayesian Poisson Tree Processes (bPTP)^44^ and Automatic Barcode Gap Discovery (ABGD)^45^ are widely used. These approaches have been widely applied to distinguish or resolve taxonomic status of the specimen by several researchers^5,25–27,46–48^. There is no comprehensive molecular assessment of fishes of river systems, in particular, Brahmaputra, Barak and Chindwin, located in the states of Meghalaya, Manipur, Mizoram and Nagaland. In the background of the above facts, this study was carried out with a view to establish a DNA barcode library and genetic diversity analyses for fish-fauna of four NE states of India (part of Indo-Myanmar Biodiversity hotspot). This study substantially aids in our understanding of fish diversity of the river systems of north-east India. Further, information generated through this study will provide an adequate knowledge base to non taxonomists, researchers, biodiversity managers and policy makers to sketch out effective conservation measures for this ecosystem.

## Result

A total of 363 individuals, representing 109 morphologically identified species, were barcoded by amplification and nucleotide sequencing of partial 5′ region of the COI mitochondrial gene. The entire dataset of 363 COI sequences was represented by 109 species, 57 genera, 22 family and 7 orders. Out of 109 species, 37 were represented by single specimens. We were not able to generate the good quality sequence reads for some of the species even after repeated attempts. Therefore, these species were excluded from the final analyses. All other amplified sequences were of >600 bp with no deletion, insertion or stop codon, indicating that they represented functional mitochondrial COI sequences. Multiple sequences were analyzed, in the studied species, to infer per cent intra-specific divergence (mean = 3.33 specimens per species, range 1–22). Nucleotide diversity of the entire dataset was found to be 0.19327 and a total of 328 polymorphic sites were identified. Parsimony informative sites with two, three and four variants were found to be 314, 38 and 129, respectively. A total number of 184 haplotype with diversity of 0.9924 were also identified in complete dataset. Overall nucleotide composition and mean GC content at codon position 1–3 is given in Table 1. Overall GC% was calculated to be 45.6 and the highest GC% of 47.8 was recorded in the fishes, representing order perciformes. All the generated barcodes were deposited in GenBank and accession numbers were obtained. Details about the species collected, number of individuals, IUCN red list status and GenBank Accession Number are furnished in Supplementary Table 1. A total of 16 DNA barcodes, previously unreported from nine species, were also generated in the present study. This includes barcodes of *Semiplotus manipurensis* (MG736433), *Paracanthocobitis adelaidae* (KX576654), *Schistura naganesis* (KU681461), *S. manipurensis* (MG736508), *Lepidocephalicthys berdmorei* (KX886802, MG778696), *Mystus cineracius* (KX886803, MG736520-21.), *Akysis manipurensis* (KX886801, MG736543), *Pillaia indica* (KJ936644-47) and *Badis ferrarisi* (KX886804). In addition to that, 11 new barcodes of seven species (un-described and identified up-to generic level only) were also generated, representing genus *Garra* (MG736488-92), *Paracanthocobitis* (MG736495), *Schistura* (MG736506-07), *Ompok* (MG736537), *Glyptothorax* (MG736554) and *Badis* (MG736580).

**Table 1:** Nucleotide composition, overall and order wise GC content and GC at codon position 1, 2 & 3.

Sl. No.	Nucleotide	All species (%)	Order-wise (%)						
			Ost	Ang	Clu	Cyp	Slu	Sym	Per
1	G	18.0	17.4	18.2	19.2	17.9	17.9	17.9	18.4
2	C	27.6	26.2	26.9	27.7	27.1	27.5	28.3	29.4
3	A	25.7	27.7	26.2	23.3	26.2	25.9	26.0	23.4
4	T	28.7	28.7	28.7	29.8	28.8	28.7	27.8	28.7
5	**GC**	**45.6**	**43.6**	**45.1**	**46.9**	**45.0**	**45.4**	**46.2**	**47.8**
6	GC1	44.0	43.4	43.9	43.9	44.2	43.9	44.0	43.7
7	GC2	36.0	31.2	33.7	39.5	34.2	35.0	37.6	44.0
8	GC3	56.7	56.4	57.9	57.4	56.8	57.3	57.2	55.9

Ost: Osteoglossiformes, Ang: Anguilliformes, Clu: Clupeiformes, Cyp: Cypriniformes, Slu: Siluriformes, Sym: Symbranchiformes, Per: Perciformes.

### Mean genetic divergence (K2P) analysis and NJ tree

Most of the morphologically identified species of the present study were well differentiated by COI sequences. As expected, a hierarchical increase in the mean Kimura 2-Parameter (K2P) genetic divergence with increasing taxonomic levels from within a species (0.42%), to within genus (10.19%), to within families (12.77%), to within orders (19.21%) was observed (Table 2). The minimum mean K2P con-generic divergence (1.72%, SE ± 0.3) was found to be almost 4 fold higher than the average con-specific (0.42%, SE ± 0.1) divergence suggesting the presence of distinct species boundaries among the studied species. The lowest mean inter-specific divergence was observed among the *Devario* species (1.72%, SE ± 0.3) and the highest among the *Channa* species (17.35%, SE ± 1.0). NJ tree, obtained from complete COI sequence dataset, contained 117 species clusters, including the singleton species with a high level of resolution between the species, supported by high bootstrap value (Supplementary Fig. 1). Individuals of six species, namely *Neolissocheilus hexastichus, Labeo bata, L. dyocheilus, S. khugae, B. assamensis* and *Channa orientalis* formed distinct clusters, supported by high bootstrap values, indicating high intra-species divergence and possibly the presence of hidden diversity.

**Table 2:** Mean K2P distance value within various taxonomic levels (n = no. of individuals, SE = Standard Error).

Taxonomic Level	Taxa/n	Min (%)	Mean (%)	Max (%)	SE ± (%)
Within species	72/327	0.00	**0.42**	3.28	0.2
Within genus	20/242	1.72	**10.19**	17.35	0.8
Within family	12/340	4.78	**12.77**	17.87	0.8
Within order	4/358	15.75	**19.21**	23.15	1.2

### ABGD Analyses for species delimitation

**Figure 1:**
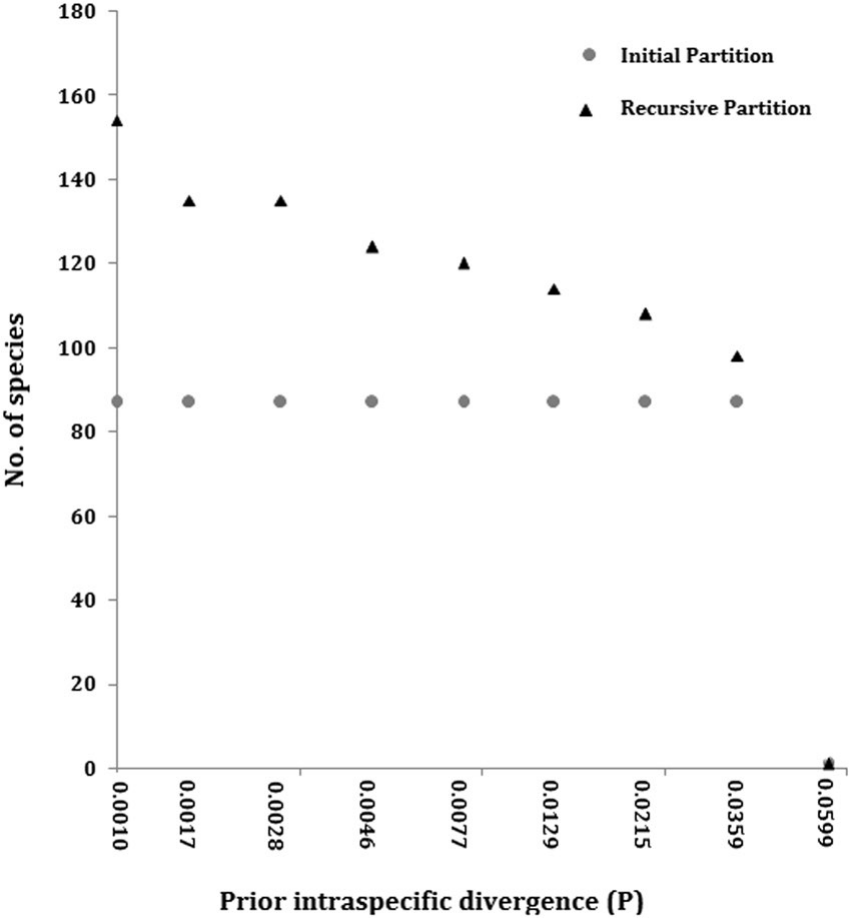
ABGD based partition of the complete data set of COI sequences of fishes of the Indo Myanmar Biodiversity hotspot. This represents the number of groups or species in each partition at different prior intra-specific divergence. The initial partition is shown as (o) and recursive partition as (∆).

To delimit the species based on single locus sequence data, species delimitation was performed in ABGD tool, using K80 Kimura distance with relative gap width (X) of 0.75 and Nb bins (for distance distribution) of 20. Partition with prior maximal distance, i.e. P = 0.0215 and 0.0129 delimited the entire dataset into 108 and 114 putative species, respectively (Fig. 1). Out of 109 morphologically identified species, 100 (90.83%) were delimited nicely through ABGD tool at a prior maximal distance of 0.0215 and were found concordant with the observations of genetic distance and NJ analysis. Further, at a prior maximal distance of 0.0215, few species of genus *Devario, Neolissocheilus, Garra* and *Mystus* could not be delimited into different putative species. Although pair-wise genetic distance and NJ analysis separated them clearly. On the other hand, few specimens of morphologically identified species *N. hexastichus, L. bata, L. dyocheilus, S. khugae, B. assamensis*, and *C. orienlatis* were delimited into two or more putative species in ABGD tool which was also confirmed by pair wise K2P distance estimation and NJ analysis.

### bPTP analysis for species delimitation

All the species delimitation results i.e. K2P genetic divergence, NJ and ABGD analyses, presented above, are based on genetic distance method. In order to further validate the distance based observation, we performed tree based species delimitation method i.e. bPTP analysis. For *de novo* species delimitation, PTP model generally outperforms GYMC as well as OTU-picking methods when evolutionary distances between species are small^44^. bPTP analysis was performed with 100,000 MCMC sampling. The bPTP analyses returned with an acceptance rate of 0.93, with merges of 49,900, splits of 50,100, and estimation of a number of species between 90 and 145 with mean value of 119.58, using single locus nucleotide sequence data. The mean number of species delimited by bPTP analysis was found close to the number of species delineated by K2P (i.e. 117 putative species with >2.00% inter-specific divergence), NJ (i.e. 117 putative species with supported bootstrap value) and ABGD (114 putative species at prior maximal distance of 0.0129) analyses.

### Pair-wise intra-specific divergence

K2P pair-wise genetic divergences were determined among all the individuals of each species except species which was represented by a single individual. Pair-wise genetic divergence analyses of few individuals of *C. orientalis*, *L. bata*, *N. hexastichus*, *B. assamensis*, *L. dyocheilus* and *S. khugae* showed high intra-specific genetic divergence (>2.0%) with no or very low con-specific variation in remaining individuals of the lineage. These observations were further confirmed by NJ (bootstrap support value) & ABGD analyses (prior maximal distance) as presented in Table 3. This possibly indicated the presence of a putative new species, species complex or geographically isolated population under the lineage.

**Table 3:** High Intra-specific divergence in some of the studied taxa.

Species	No. of individuals	Intra-specific distance % (±SE)	Con-specific distance % (±SE)	Bootstrap support value %	Prior max. distance for ABGD
*Channa orientalis*	10	3.19–6.36 (0.9)	0.0–0.98 (0.2)	86–100	0.0215
*Labeo bata*	07	4.39 (0.9)	0.00	81	0.0215
*Neolissocheilus hexastichus*	22	2.7–3.7 (1.0)	0.22–0.45 (0.2)	96–100	0.0129
*Badis assamensis*	04	3.52 (0.7)	0.88 (0.3)	97	0.0215
*Labeo dyocheilus*	04	3.29 (0.7)	0.00	100	0.0215
*Schistura khugae*	04	2.16 (0.6)	0.00	100	0.0215

### Notes on Specific Taxa

#### Garra species

Phenotypic plastic is always a hindrance in taxonomic identification of many *Garra* species and the same was experienced in the present study also. A total of 25 specimens of genus *Garra* were collected in the present survey. Out of this, 15 individuals were identified as *G. annadalaei* (07), *G. lissorhynchus* (01), *G. litanensis* (01), *G. nasuta* (05) and *G. qiaojiensis* (01). Remaining 10 individuals could be identified up-to generic level only and distributed into three morphologically distinct clusters. These individuals were designated as *Garra* sp._01 (MG7364483-87), *Garra* sp._02 (MG736488-90) and *Garra* sp._03 (MG736491-92). Homology search of COI sequence of *Garra* sp._01 showed 99% similarity with *Garra* sp. 8162 C NBFGRMU (KT896683) and *Garra* sp. Tuirivang (KF318331). The average pair-wise genetic distance, estimated between COI sequences of *Garra* sp.1, *Garra* sp. 8162 C NBFGRMU and *Garra* sp. Tuirivang, was found to be 0.56% (SE ± 0.2), revealing that they belonged to the same species which were also collected from the same geographical area and identified up-to generic level only. Specimens of *Garra* sp._02 were found to be morphologically close, but distinct from *G. naganensis*. A high genetic divergence, i.e. 3.55% (SE ± 1.0) was estimated between the individuals of *Garra* sp._02 and sister species *G. naganensis* (KX951812) which was found to be higher than the maximum conspecific pair-wise divergence (3.28%, SE ± 1.0), estimated between all the species examined in the present study. Therefore, *Garra* sp. 2 represents a putative previously undescribed species. Same analyses were also conducted for *Garra* sp. 3 which was morphologically close, but distinct from *G. mcclellandi* (KX239495). The average pair-wise genetic distance was estimated between *Garra* sp._03 and *G. mcclellandi* and was observed to be 11.31% (SE ± 2.0) whereas no inter-generic distance among *Garra* sp._03 individual was observed. The observed genetic divergence was found to be almost three and half times higher than the maximum conspecific pair-wise divergence, estimated between all the species examined.

**Figure 2:**
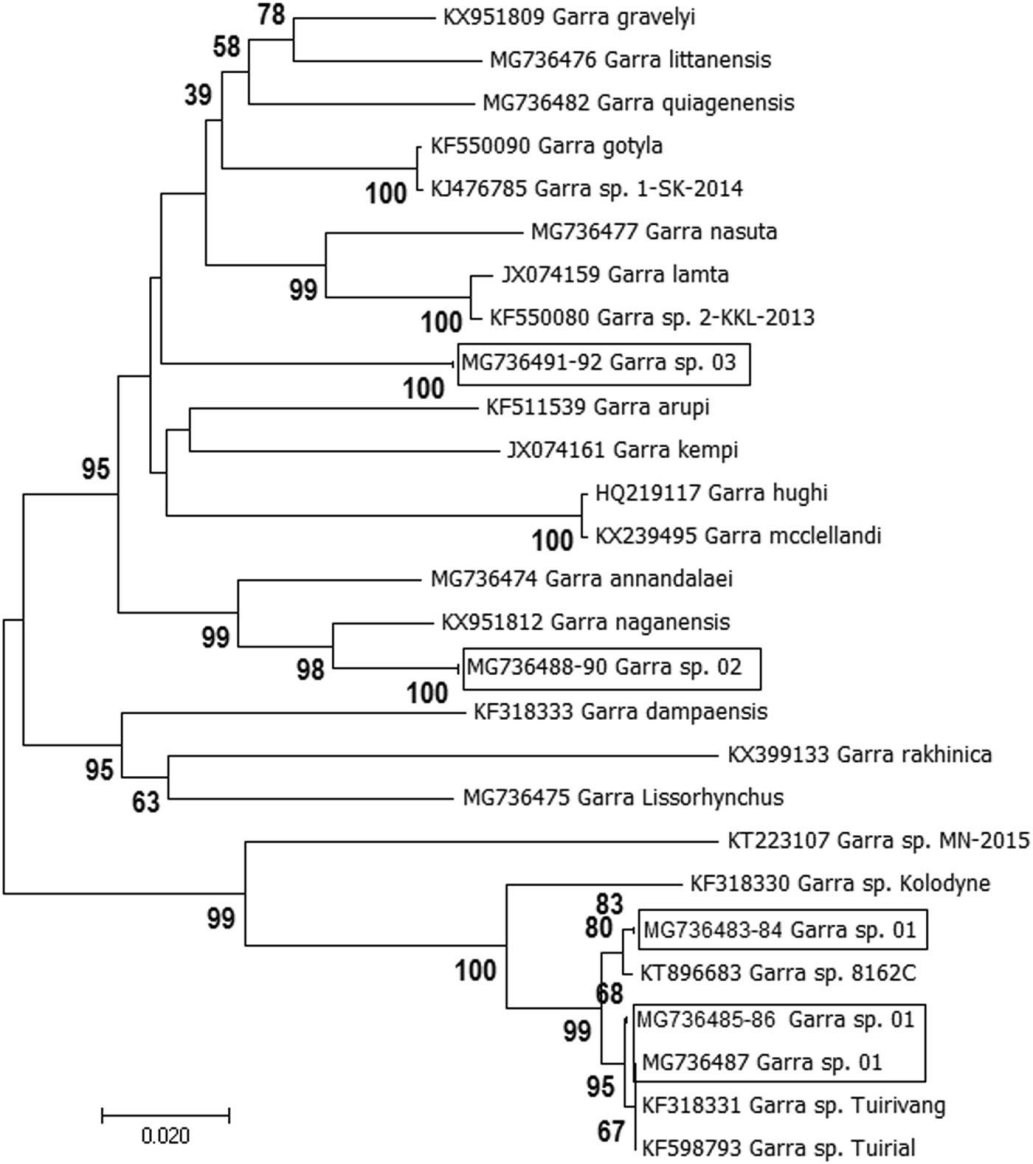
NJ analyses (based on K2P genetic distance of COI sequences) of *Garra* species (COI sequences of undescribed species are inside the box).

We further investigated to find out whether these are truly undescribed species. For this, we analyzed COI sequences of 22 *Garra* species, reported from the same or neighboring rivers and estimated pair-wise genetic divergence, followed by NJ and ABGD analyses. Out of these 22 species, COI sequence of five species, namely *G. annandalaei*, *G. lissorhynchus, G. litanensis, G. nasuta* and *G. giaojiensis* were generated in the present study whereas sequences of remaining 17 species were retrieved from NCBI database (species name & Accession no. is given in Fig. 2). NJ analyses also showed the clustering of *Garra* sp._01 with *Garra* sp. 8162 C NBFGRMU (KT896683) and *Garra* sp. Tuirivang (KF318331) with the highest supported bootstrap value (100%), suggesting that these individuals belonged to the same species. Individuals of *Garra* sp._02 were clustered separately from *G. naganensis* with the highest bootstrap value (100%). In same manner individuals of *Garra* sp._03 were also grouped separately from *G. mccellandi* with the supported bootstrap value. ABGD analyses at a prior maximal distance of 0.0215 also delineated *Garra* sp._02 and *Garra* sp._03 into a separate partition. These findings support that *Garra* sp._02 and *Garra* sp._03, collected in the present study, represents a putative and previously undescribed species.

#### Paracanthocobitis species

**Figure 3:**
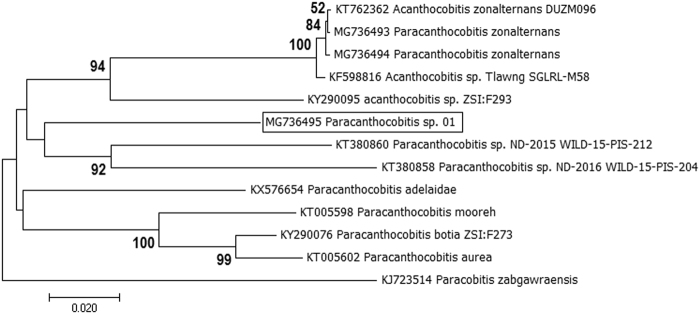
NJ analyses (based on K2P genetic distance of COI sequences) of *Paracanthocobitis* species (COI sequences of undescribed species is inside the box).

One specimen of *Paracanthocobitis* species which was morphologically close, but distinct from *Paracanthocobitis botia* appeared to be unrecognized and designated as *Paracanthocobitis* sp._01 (MG736495). We further investigated to find out whether this is a truly undescribed species. For this, we analyzed the COI sequences of 10 species of *Paracanthocobitis*, available in the NCBI GenBank Database and reported from Indian and neighboring river (Species Name & Accession no. provided in Fig. 3). *Paracanthocobitis* sp._01 showed very high genetic divergence with the sister species *Paracanthocobitis botia, i.e*. 12.89%, SE ± 0.2 which was almost four times more than the maximum conspecific pair-wise divergence (3.28%, SE ± 1.0), estimated between all the species, examined in the present study. Average distance with other studied species of *Paracanthocobitis* was recorded as 14.25%, SE ± 2.0. NJ analyses also showed separate clustering of *Paracanthocobitis* sp._01. ABGD analyses at a prior maximal distance of 0.0215 also delineated this species into a separate partition. These findings support that *Paracanthocobitis* sp._01, collected in the present study, represents a putative and previously undescribed species.

#### Schistura species

**Figure 4:**
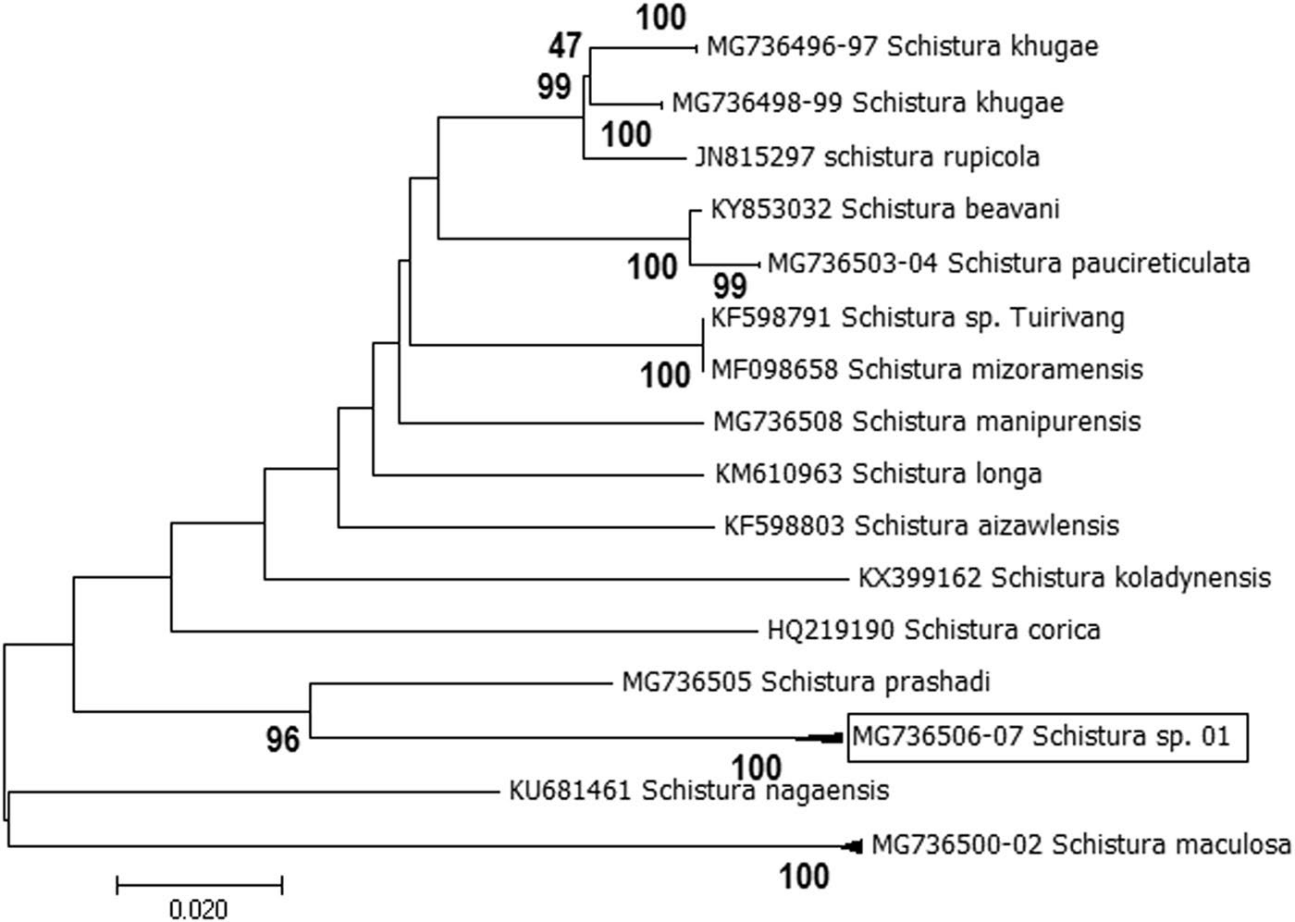
NJ analyses (based on K2P genetic distance of COI sequences) of *Schistura* species (COI sequences of undescribed species are inside the box).

A total of 12 individuals of *Schistura* Species were collected and out of which 10 individuals were assigned as *S. khugae* (04), *S. maculosa* (03), *S. manipurensis* (01), *S. naganesis* (01), and *S. prashadi* (01). Remaining two individuals of *Schistura* species could be identified up-to genus level and designated as *Schistura* sp._01 (MG736506-07). We compared the sequence of *Schistura* sp._01 with 14 other species of *Schistura*, reported from same or neighboring rivers. Out of 14 species, COI sequences of six species namely *S. khugae, S. maculosa, S. manipurensis, S. nagaensis, S. paucireticulata* and *S. prashadi* were generated in the present study, whereas the sequences of remaining eight species were downloaded from the NCBI database (species name & Genbank Accession no. is given in Fig. 4). The closest genetic distance was observed with *S. prashadi* i.e. 9.55% (SE ± 2.0) which was found almost three times higher than the maximum conspecific pair-wise divergence (3.28%, SE ± 1.0), estimated between all the species examined. Average distance with other studied species of *Schistura* was recorded as 16.68% (SE ± 2.0). NJ analyses also showed separate clustering of *Schistura* sp._01 with high bootstrap support value i.e. 96.0%. ABGD analyses at a prior maximal distance of 0.0215 also delineated this species into a separate partition. These findings support that *Schistura* sp._01, collected in the present study, represents a putative and previously undescribed species.

#### Ompok Species

One specimen of species *Ompok*, which was morphologically close, but distinct from *Ompok pabda* appeared to be unrecognized and designated as *Ompok* sp._01 (MG736537). We further investigated to find out whether this is a truly undescribed species. For this we analysed COI sequences of all the valid species of *Ompok*, reported from Indian water i.e. *O. pabda, O*. *pabo, O. bimaculatus* and *O. malabaricus* (restricted to Malabar Coast of India) except O*. karunkodu* (restricted to Amaravathi, a right-hand tributary of the River Kaveri in Southern India). Reason for excluding *O. karunkodu* from the analyses is unavailability of COI sequence in public databases. COI sequences of analysed species, namely *O. pabda* (KT762383), *O. pabo* (FJ230020), *O. bimaculatus* (FJ230051), and *O. malabaricus* (HQ009495) were retrieved from the NCBI GenBank database. *Ompok* sp. 1 showed very high genetic divergence with the sister species *O. pabda i.e*. 11.47%, SE ± 2.0 which was almost three and half times more than the maximum conspecific pair-wise divergence (3.28%, SE ± 1.0), estimated between all the species examined in the present study. Average distance with other studied species of *Ompok* was recorded as 13.39%, SE ± 2.0. NJ analyses also showed separate clustering of *Ompok* sp._01 with bootstrap value of 99.0%. ABGD analyses at a prior maximal distance of 0.0215 also delineated this species into a separate partition. These findings support that *Ompok* sp._01, collected in the present study, represents a putative and previously undescribed species.

#### Glyptothorax species

**Figure 5:**
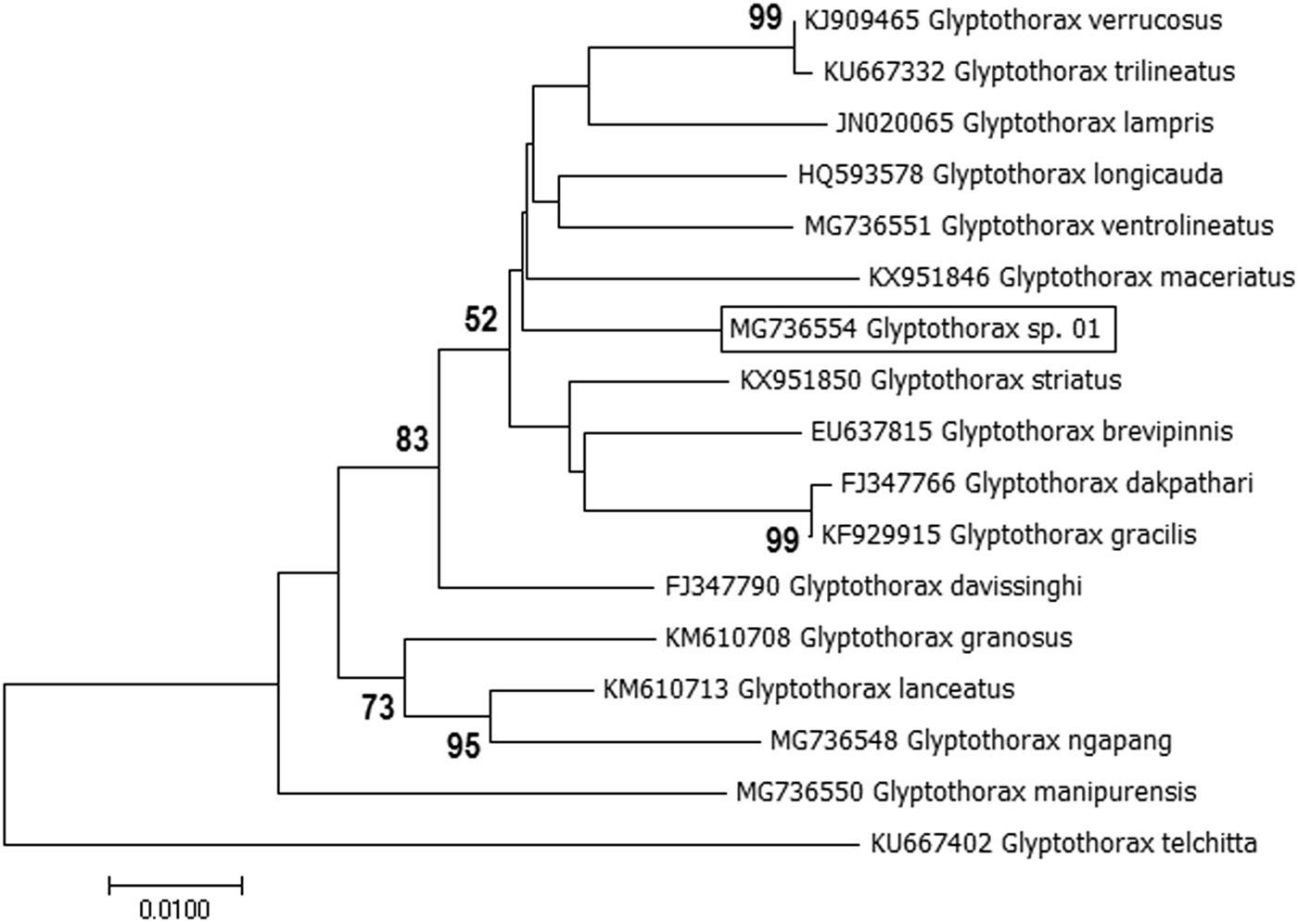
NJ analyses (based on K2P genetic distance of COI sequences) of *Glyptothorax* species (COI sequences of undescribed species is inside the box).

One specimen of genus *Glyptothorax* was found morphologically close, but distinct from *Glyptothorax verrucosus* and appeared to be unrecognized and designated as *Glyptothorax* sp._01 (MG736554). We investigated to find out whether this is a truly undescribed species. For this, we analysed COI sequences of 16 *Glyptothorax* species, reported from the same or neighboring waters (species name & Genbank Accession no. is given in Fig. 5). Out of these 16 species, COI sequence of 05 species, namely *G. ventrolineatus, G. ngapang, G. manipurensis, G. telchitta* and *G. trilineatus* were generated in the present study. *Glyptothorax* sp. 1 showed high genetic divergence with the sister species *G. verrucosus i.e*. 3.16%, SE ± 1.0. Average distance with other studied species of *Glyptothorax* was recorded as 5.79%, SE ± 1.0. NJ analyses also showed separate clustering of *Glyptothorax* sp._01 and ABGD analyses at a prior maximal distance of 0.0215 also delineated this species into a separate partition. These findings support that *Glyptothorax* sp._01, collected in the present study, represents a putative previously un-described species.

#### Badis species

**Figure 6:**
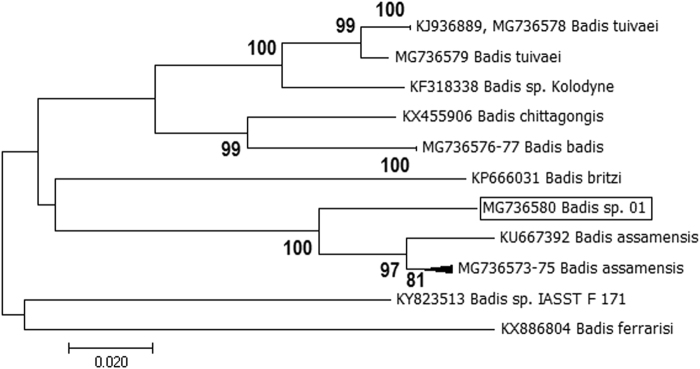
NJ analyses (based on K2P genetic distance of COI sequences) of *Badis* species (COI sequences of undescribed species is inside the box).

One specimen of genus *Badis* was found morphologically close, but distinct from *Badis assamensis* and appeared to be unrecognized and designated as *Badis* sp._01 (MG736580). We investigated to find out whether this is a truly un-described species. For this, we analyzed COI sequences of 08 species of *Badis*, reported from the same or neighboring waters (species name & Genbank Accession no. is given in Fig. 6). Sequence of *B. assamensis*, *B. badis*, *B. ferrarisi* and *B. tuivaei* were generated in the present study. *Badis* sp._01 showed high genetic divergence with the sister species *B. assamensis i.e*. 5.87%, SE ± 2.0 which was found higher than the maximum conspecific pair-wise divergence (3.28%, SE ± 1.0), estimated between all the species examined. Average distance with other studied species of *Badis* was recorded as 16.04%, SE ± 1.0. NJ analyses also showed separate clustering of *Badis* sp._01 with the highest bootstrap support value of 100%. ABGD analyses at a prior maximal distance of 0.0215 also delineated this species into a separate partition. These findings support that *Badis* sp._01, collected in the present study, represents a putative previously un-described species.

### Note on distribution record

The collection sites of majority of the species, studied in the present report, were found concordant with their earlier distribution record. Although, some species, recorded from new locations (not reported earlier), are presented here. In the present study, a species namely, *Devario deruptotalea*, earlier reported only from the tributary of the Yu River (Chindwin drainage) in Manipur, India was also collected from river Tuphaleri of Nagaland. Another species *Osteobrama feae*, which was reported only from Myanmar & China, was also collected from a tributary of Chindwin in Manipur. One exotic species, *Barbonymus gonionotus*, with its natural distribution in the Mekong and Chao Phraya basins, Malay Peninsula, Sumatra and Java, was also reported from Mizoram and Meghalaya, which indicates the escape of this species from culture installations to natural water bodies. Another exotic species, *Ctenopharyngodon idella* (grass carp), was recorded from Meghalaya, indicating the escape from culture system to the river. *Hypsibarbus myitkyinae*, reported only from Myanmar, was also collected from the tributary of Chindwin from Manipur. Another species *Schizothorax molesworthi*, known from the Brahmaputra River drainage was also recorded from Chindwin drainage of Nagaland. A recently described species *Schistura paucireticulata* from Tuirial River, a major tributary of the Barak River of Mizoram, was also collected from the Tlwang River of Mizoram^49^. *Mystus cineraceus* and *B. ferrarisi* described only from Irrawaddy drainage of Myanmar, was also recorded from Manipur. Few endemic species, described and reported from northeast India, were also collected from their type locality or nearby in the present study and are presented here. A miniature catfish *Pseudolaguvia virgulata*, described and reported from the Barak River drainage in Mizoram, was also collected from a nearby locality. A hill stream spineless eel *Pillaia indica* (reported from Garo Hills of Meghalaya) and freshwater glass perch *Parambassis waikhomi* (described and reported from Loktak Lake of Manipur) were also collected from their type locality in the present study.

## Discussion

Species are considered as a basic unit of biodiversity and is the foundation of ecosystem services to which human well-being is closely linked. Therefore, the precise answer on biodiversity is needed to devise an effective measure to conserve biodiversity (Source: Millennium Ecosystem Assessment: Ecosystems and Human Well-being: Biodiversity Synthesis, 2005). Indo-Myanmar Biodiversity hotspot represents the area of high endemism which is under continuous pressure^1^. Therefore, the need of the present study was realized by the authors which deals with the first comprehensive molecular assessment of fish fauna of four states (i.e. Mizoram, Meghalaya, Manipur and Nagaland and comes under the biodiversity hotspot) of northeast India. This study includes the molecular identification of 109 species and development of the DNA barcode reference library which will provide efficient and easy identification system for fish fauna of the region. These 109 species includes 46.36% of the reported fish fauna of the region as per the Records of Zoological Survey of India^2^.

We observed hierarchical increase in mean K2P genetic divergence (%) with increasing taxonomic level (0.42, 10.19, 12.77 & 19.21) which is almost similar to the previous reports, documented on the freshwater fishes of India^5,25,50,51^, Canada^52^ and Salween river of southwestern China^26^. Observations were also found similar for Indian marine fishes^53,54^. Earlier studies have attempted to use barcoding data alone for delineating the species boundaries^20,55–57^. Hebert and co-workers tried to establish a standard COI sequence threshold between conspecific and congeneric divergence i.e. also called as 10 X rule where 10 folds of mean intraspecific variation are adequate to draw the species boundary^58^. In the present study, 10 X rule was not found to be fit for delineating the species which was also in accordance with few previous reports^55,59^. Further, frequent overlap between intraspecific and interspecific divergence has been reported in earlier studies, thus, generalizing the threshold for species level resolution has been considered difficult. Therefore, combined use of mean K2P genetic divergence and various species delimitation tools are more effective for delineating the species and defining the boundaries^5,25,26,47,60^.

The estimated number of species using ABGD model was found to be 108 and based on this value 90.83% of the studied species were delineated nicely. However, some taxa like *N. hexastichus, L. bata, L. dyocheilus, B. assamensis, S. khugae, C. orientalis, Garra* sp._01-03, *Paracanthocobitis* sp._01*, Schistura* sp._01, *Ompok* sp._01*, Glyptothorax* sp._01, and *Badis* sp._01 which did not show clear relationship and high divergence value, were also partitioned well with the help of NJ and ABGD species delineation analyses. The species which were not delineated properly through ABGD tool at a distance of 0.0215, were further separated at a low proximal distance of 0.0129, followed by confirmation using pair-wise genetic distance and NJ analysis. Tree based species delimitation i.e. bPTP, resulted into 119 number of species (mean value) which was found closed to the number of species delineated by K2P (i.e. 117 putative species with >2.00% inter-specific divergence), NJ (i.e. 117 putative species with supported bootstrap value) and ABGD (114 putative species at prior maximal distance of 0.0129) analyses. Therefore, the combination of genetic divergence along with NJ, ABGD & bPTP analyses may help in correct identification of the fish fauna of the region.

A total of 291 species has been reported from the entire northeast India as per the Records of Zoological Survey of India^2^. Out of these, 241 species were reported from Mizoram, Manipur, Meghalaya and Nagaland. Although, drainage-wise exploration studies are scanty in the region, but some workers like Vishwanath *et al*. reported 117 fish species in Chindwin drainage flowing in Manipur^3^. Kar & Sen reported 151 fish fauna from Tripura, Barak drainage flows in parts of Assam, Manipur and Mizoram and Karnafuli and Koladyne/Kaladan drainage of Mizoram^4^. Barman *et al*. also reported 51 fish species from the Kaladan River system, flowing in Mizoram^5^. Four species, namely *Tor putitora*, *P. manipurensis*, *P. indica* and *B. tuivaei*, collected in present study, are included under IUCN Red list (2014) category of endangered species whereas seven species, namely, *B. ngawa*, *P. atra*, *G. litanensis*, *S. khugae*, *S. nagaensis*, *S. prashadi* and *G*. *manipurensis* under the category of vulnerable species and nine species, namely, *A. bengalensis*, *N. hexagonolepis*, *N. hexastichus*, *O. belangeri*, *S. manipurensis*, *O. bimaculatus*, *O. pabda* and *W. attu* under the category of near threatened species. Majority of the species which are included under the category of endangered, vulnerable and near threatened have sporadic distribution. The sporadic distribution of most of the above mentioned species indicated a decline in the population. Although, this interpretation must be considered very cautiously because inadequate sampling cannot be ruled out in the present study. Variations in terms of number and type of species in comparison to previous studies can be explained by the cryptic diversity, phenotypic plasticity, misidentification, and taxonomic ambiguities.

In conclusion, it is confirmed that the integrated use of DNA barcoding (genetic divergence, NJ, ABGD & bPTP species delineation) and morphological evidences are efficient and reliable tools for identification of fishes at the species level. Further, establishment of a reliable COI barcode database of fish fauna of the Indo-Myanmar region may serve as a reference library for accurate identification of fishes that will surely help ichthyologists, researchers, students, biodiversity managers and policy makers. Presence of high degree of pair-wise intra-specific divergence among the individuals of some species was revealed, suggesting the presence of putative sibling species or existence of hidden diversity among the fish species, advocating the immediate need for more comprehensive studies on fish fauna of the region.

## Materials and Methods

### Ethical statement

The fishes, studied in the present study, are not protected under The Wildlife Protection Act, 1972 (Last amended in 2013), Government of India and caught with the help of local fisherman. No experimentation was conducted on live specimens in the laboratory and study was approved by the Institutional Animal Ethics Committee (IAEC) of College of Fisheries (Central Agricultural University, Imphal), India.

### Sample collection and DNA isolation

Fishes were collected from 40 distant locations of different rivers and tributaries, covering four states of northeast India (an essential part of Indo-Myanmar Biodiversity Hotspot). A total of 109 morphologically identified species were collected during August 2013 to February 2017 from different locations of Meghalaya, Manipur, Mizoram and Nagaland. Information about the sampling stations along with geographical coordinates is given in Supplementary Table 2. Present study includes fishes from Barak River and its tributaries, flowing in Mizoram and Manipur, tributaries of Brahmaputra River flowing in Nagaland and Meghalaya, tributaries of Chindwin river system flowing in Manipur and Nagaland and Karnafuli river of Mizoram.

For species identification and nomenclature, information available at www.fishbase.org and taxonomic keys prepared by various taxonomists were followed^7,49,61–74^. The representative voucher specimen of all the collected species except *Garra qiaojiensis, Lepidocephalichthys annandalei, Wallago attu* and *Paracanthocobitis zonalternans* are maintained at the museum, established with partial financial assistance given by the Department of Biotechnology, Government of India under the center of excellence project at the College of Fisheries (Central Agricultural University), Tripura, India. Approximately 100 mg of white muscle tissue or 200-500 μl of whole blood from each specimen were preserved in 95% ethanol for genomic DNA isolation. Total genomic DNA was extracted from the muscle tissue or whole blood, using the standard phenol-chloroform-isoamyl-alcohol method described by Sambrook and Russell with some minor modifications^75^. The integrity of the isolated samples was determined by running the samples in 0.8% agarose gel, stained with ethidium bromide. Concentration and purity of isolated samples were determined with the help of nano-volume spectrophotometer (mySpec Scientific GmbH) and stored at −20 °C for further use.

### COI Amplification and purification

For DNA barcoding, partial 5′ region of COI gene was amplified in a final volume of 50 μl with final concentration of 1X reaction buffer (10 mM Tris-HCl _pH 8.3_, 50 mM KCl,), 2.0 mM MgCl_2_, 0.2 mM of dNTP mix, 10 pmol of forward and reverse primer, 2U of Taq DNA polymerase and 100 ng of template DNA. The primers used for amplification of COI gene were 5′-TCAACCA ACCACAAAGACATTGGCAC-3′ and 5′-TAGACTTCTGGGTGGCCAAAGAATCA-3′^76^. Each reaction included a negative control (no template DNA) and was carried out in an Veriti 96 well thermal cycler of Applied Biosystems under following thermal cycling conditions: initial denaturation at 95 °C for 4 min, followed by 35 cycles of denaturation at 94 °C for 35 sec, primer annealing at 52 °C for 30 sec and primer extension at 72 °C for 40 sec and final extension for 5 min at 72 °C. Amplified products of COI were separated on 1.5% agarose gel, stained with ethidium bromide, followed by gel elution using spin column based gel extraction kit of Thermo Fisher as per the manufacturer’s instructions. In the final step, purified products were eluted in 1X TE buffer.

### Nucleotide Sequencing of COI gene

The purified products (free of salts and protein impurities) of COI were directly sequenced in Applied Biosystem 3500 genetic analyzer using BigDye^®^ Terminator Cycle Sequencing Kit (Applied Biosystems) as per manufacturer’s instructions. The sequencing (dye labeling) reaction was optimized to a final volume of 10 μl, using 50 ng template DNA, 33 μM primer, 1X BigDye Terminator reaction mix, followed by thermal cycling at initial denaturation at 96 °C for 4 min, 25 cycles of denaturation at 94 °C for 10 sec, annealing at 50 °C for 10 sec and extension at 60 °C for 4 min. The labeled products were precipitated with 0.1 volume of 3 M sodium acetate (pH 4.6) and 2.5 volumes of ethanol, followed by centrifugation at room temperature for 30 min at 14,000 × g. The pelleted products were washed with 70% ethanol and air dried, followed by resuspension in 15 μl of Hi-Di formamide solution. The samples were denatured at 95 °C for 5 min and chilled on ice for 5 min to prevent the renaturation. The denatured samples were transferred into 96 well sequencing plate with rubber closure and placed into ABI 3500 genetic analyzer for nucleotide sequencing at a DNA sequencing facility of central molecular biology laboratory of the College of Fisheries (CAU, Imphal), India.

### Genetic diversity analysis and phylogenetic tree construction

To rule out sequencing error, bidirectional 2X overage were performed (each base sequenced 2 times) for each studied sample. Chromatograms of each sequence were examined carefully to verify all base pair assignments, using ABI sequence viewing software. All the COI sequences of mtDNA region were assembled and aligned, using MEGA 7, followed by determination of nucleotide composition^77^. DnaSp ver 5.10 was used to estimate the number of polymorphic sites and nucleotide diversity (Pi)^78^. Pair-wise and mean K2P genetic distance was calculated with the help of MEGA 7. Neighbor-joining (NJ) approach was employed to deduce phylogenetic relationships among species, using COI sequences with the help of MEGA 7 and support for monophyly was assessed with 10000 bootstrap pseudo-replicates^79^. To delimit the species based on single locus sequence data, species delimitation was performed in ABGD tool, using JC69 Jukes-Cantor distance with relative gap width (X) of 0.75 and Nb bins (for distance distribution) of 20^45^. Tree based species delimition was also performed to validate the observations made by distance based delimitation method^44^. For this, RAxML majority rule extended consensus (MRE) tree was obtained from unique haplotype sequences^80^. The obtained MRE tree was imported to bPTP web server and analysis was performed with 100,000 MCMC generations.

## Electronic supplementary material

Supplementary Material
