# Supplementary material for: DNA Barcoding of Freshwater Fishes of Indo-Myanmar Biodiversity Hotspot
Source: Sci Rep. 2018 Jun 5;8:8579. doi: 10.1038/s41598-018-26976-3 (PMC5988717; doi:10.1038/s41598-018-26976-3)
Supplement: Supplementary file 1 — Supplementary Material [file 41598_2018_26976_MOESM1_ESM.pdf]

Manuscript ID: SREP-18-02021

Manuscript Title: DNA Barcoding of Freshwater Fishes of Indo-Myanmar Biodiversity Hotspot

Authors: Anindya Sundar Barman*, Mamta Singh, Soibam Khogen Singh, Himadri Saha, Yumlembam Jackie Singh, Martina Laishram & Pramod Kumar Pandey

***Corresponding author**
Dr. Anindya Sundar Barman
College of Fisheries (Central Agricultural University, Imphal)
Lembucherra Tripura West (799210), INDIA
Email: barman.anindya@gmail.com
Phone No: +91-381-2865264
Fax No: +91-381-2865291

Dr. Mamta Singh
College of Fisheries (Central Agricultural University)
Lembucherra, Tripura (West), INDIA
Email: mamta0131@gmail.com

Dr. Soibam Khogen Singh
College of Fisheries (Central Agricultural University)
Lembucherra, Tripura (West), INDIA
Email: gengang@gmail.com

Dr. Himadri Saha
College of Fisheries (Central Agricultural University)
Lembucherra, Tripura (West), INDIA
Email: sahacofcau@gmail.com

Mr. Yumlembam Jackie Singh
College of Fisheries (Central Agricultural University)
Lembucherra, Tripura (West), INDIA
Email: jky.ext@gmail.com

Ms. Martina Laishram
College of Fisheries (Central Agricultural University)
Lembucherra, Tripura (West), INDIA
Email: martinalaishram41@gmail.com

Dr. Pramod Kumar Pandey
College of Fisheries (Central Agricultural University)
Lembucherra, Tripura (West), INDIA
Email: pkpandey_in@yahoo.co.uk

Supplementary Table 1: List of species collected with taxonomic details, number of individual barcoded, IUCN red list status and GenBank Accession number

Sl. No.	Species	Family	Order	No. of Individuals	IUCN Red List Status*	GenBank Accession No.
1	*Notopterus notopterus*	Notopteridae	Osteoglossiformes	03	LC	MG736322-324
2	*Anguilla bengalensis*	Anguillidae	Anguilliformes	01	NT	MG736325
3	*Gudusia chapra*	Clupeidae	Clupeiformes	02	LC	KJ936659, MG736326
4	*Barillius bendelesis*	Cyprinidae	Cypriniformes	02	LC	KJ936749-750
5	*Barillius ngawa*	Cyprinidae	Cypriniformes	04	VU	MG736327-330
6	*Barillius vagra*	Cyprinidae	Cypriniformes	01	LC	MG736331
7	*Esomus danricus*	Cyprinidae	Cypriniformes	05	LC	KJ936754-58
8	*Danio meghalayensis*	Cyprinidae	Cypriniformes	01	NE	MG736332
9	*Danio rerio*	Cyprinidae	Cypriniformes	01	LC	MG736333
10	*Devario aequipinnatus*	Cyprinidae	Cypriniformes	21	LC	KJ936784-788, MG736334-349
11	*Devario deruptotalea*	Cyprinidae	Cypriniformes	02	NE	MG736350-351
12	*Amblypharangodon mola*	Cyprinidae	Cypriniformes	07	LC	KJ936759-63, KJ936815-16
13	*Ctenopharyngodon idella*	Cyprinidae	Cypriniformes	01	NE	MG736352
14	*Tor putitora*	Cyprinidae	Cypriniformes	02	EN	MG736353-354
15	*Neolissochilus hexagonolepis*	Cyprinidae	Cypriniformes	05	NT	MG736355-359
16	*Neolissochilus hexasticus*	Cyprinidae	Cypriniformes	22	NT	KJ936794-797, MG736360-377
17	*Neolissochilus stracheyi*	Cyprinidae	Cypriniformes	07	LC	MG736378-384
18	*Osteobrama belangeri*	Cyprinidae	Cypriniformes	02	NT	MG736385-386
19	*Osteobrama feae*	Cyprinidae	Cypriniformes	02	LC	MG736387-388
20	*Chagunius chagunio*	Cyprinidae	Cypriniformes	01	LC	MG736389
21	*Puntius chola*	Cyprinidae	Cypriniformes	06	LC	KJ936779, MG736390-394
22	*Puntius sophore*	Cyprinidae	Cypriniformes	06	LC	MG736395-400
23	*Pethia atra*	Cyprinidae	Cypriniformes	03	VU	MG736401-403
24	*Pethia conchonius*	Cyprinidae	Cypriniformes	01	LC	MG736404
25	*Pethia manipurensis*	Cyprinidae	Cypriniformes	02	EN	MG736405-406
26	*Barbonymus gonionotus*	Cyprinidae	Cypriniformes	10	LC	KJ936769-773, MG736407-411
27	*Hypsibarbous myitkiyae*	Cyprinidae	Cypriniformes	21	LC	MG736412-432
28	*Semiplotus manipurensis.*	Cyprinidae	Cypriniformes	01	DD	MG736433
29	*Cirrhinus mrigala*	Cyprinidae	Cypriniformes	05	LC	KJ936734-735, MG736434-436 [illegible]
30	*Gebelion catla*	Cyprinidae	Cypriniformes	01	LC	MG736437
31	*Labeo bata*	Cyprinidae	Cypriniformes	07	LC	MG736438-444
32	*Labeo calbasu*	Cyprinidae	Cypriniformes	04	LC	KJ936890-891, MG736445-446
33	*Labeo dyochielus*	Cyprinidae	Cypriniformes	04	LC	MG736447-450
34	*Labeo gonius*	Cyprinidae	Cypriniformes	02	LC	MG736451-452
35	*Labeo rohita*	Cyprinidae	Cypriniformes	04	LC	MG736453-456
36	*Bangana ariza*	Cyprinidae	Cypriniformes	02	LC	MG736457-458
37	*Bangana dero*	Cyprinidae	Cypriniformes	04	LC	MG736459-462
38	*Schizothorax molesworthi*	Cyprinidae	Cypriniformes	01	DD	MG736463
39	*Crossocheilus latius*	Cyprinidae	Cypriniformes	06	LC	MG736464-469
40	*Garra annandalei*	Cyprinidae	Cypriniformes	07	LC	KJ936806-807, MG736470-474
41	*Garra lissorhynchus*	Cyprinidae	Cypriniformes	01	LC	MG736475

42	*Garra litanensis*	Cyprinidae	Cypriniformes	**01**	**VU**	MG736476
43	*Garra nasuta*	Cyprinidae	Cypriniformes	**05**	**LC**	MG736477-481
44	*Garra qiaojiensis*	Cyprinidae	Cypriniformes	**01**	**DD**	MG736482
45	*Garra* sp._01	Cyprinidae	Cypriniformes	**05**	**--**	MG736483-487
46	*Garra* sp._02	Cyprinidae	Cypriniformes	**03**	**--**	MG736488-490
47	*Garra* sp._03	Cyprinidae	Cypriniformes	**02**	**--**	MG736491-492
48	*Psilorhynchus sucatio*	Psilorhynchidae	Cypriniformes	**01**	**LC**	KU667397
49	*Paracanthocobitis adelaideae*	Balitoridae	Cypriniformes	**01**	**NE**	KX576654
50	*Paracanthocobitis zonalternans*	Balitoridae	Cypriniformes	**02**	**LC**	MG736493-494
51	*Paracanthocobitis* sp. _01	Balitoridae	Cypriniformes	**01**	**-**	MG736495
52	*Schistura khugae*	Balitoridae	Cypriniformes	**04**	**VU**	MG736496-499
53	*Schistura maculosa*	Balitoridae	Cypriniformes	**03**	**NE**	MG736500-502
54	*Schistura manipurensis*	Balitoridae	Cypriniformes	**01**	**NT**	MG736508
55	*Schistura nagaensis*	Balitoridae	Cypriniformes	**01**	**VU**	KU681461
56	*Schistura paucireticulata*	Balitoridae	Cypriniformes	**02**	**NE**	MG736503-504
57	*Schistura prashadi*	Balitoridae	Cypriniformes	**01**	**VU**	MG736505
58	*Schistura* sp. _01	Balitoridae	Cypriniformes	**02**	**-**	MG736506-507
59	*Syncrossus berdmorei*	Cobitidae	Cypriniformes	**01**	**NT**	MG736509
60	*Botia dario*	Cobitidae	Cypriniformes	**02**	**LC**	MG736510-511
61	*Lepidocephalichthys annandalei*	Cobitidae	Cypriniformes	**01**	**LC**	MG736512
62	*Lepidocephalichthys berdmorei*	Cobitidae	Cypriniformes	**02**	**LC**	KX886802, MG778696
63	*Lepidocephalichthys guntea*	Cobitidae	Cypriniformes	**07**	**LC**	KJ936714, MG736513-518
64	*Acantopsis choirorhynchos*	Cobitidae	Cypriniformes	**01**	**LC**	MG736519
65	*Sperata seengala*	Bagridae	Siluriformes	**01**	**LC**	KJ936840
66	*Mystus bleekeri*	Bagridae	Siluriformes	**05**	**LC**	KJ936764-766, KJ936689-690
67	*Mystus cineraceus*	Bagridae	Siluriformes	**03**	**DD**	KX886803, MG736520-521
68	*Mystus ngasep*	Bagridae	Siluriformes	**07**	**NE**	MG736522-528
69	*Mystus vittatus*	Bagridae	Siluriformes	**01**	**LC**	KJ936694
70	*Batasio affinis*	Bagridae	Siluriformes	**02**	**DD**	MG736529-530
71	*Olyra longicadulata*	Bagridae	Siluriformes	**02**	**LC**	MG736531-532
72	*Ompok bimaculatus*	Siluridae	Siluriformes	**03**	**NT**	MG736533-535
73	*Ompok pabda*	Siluridae	Siluriformes	**01**	**NT**	MG736536
74	*Ompok* sp._01	Siluridae	Siluriformes	**01**	**-**	MG736537
75	*Wallago attu*	Siluridae	Siluriformes	**01**	**NT**	KJ936719
76	*Clupisoma prateri*	Schilbidae	Siluriformes	**01**	**LC**	MG736540
77	*Pachypterus atherinoides*	Schilbidae	Siluriformes	**02**	**LC**	MG736538-539
78	*Amblyceps apangi*	Amblycipitidae	Siluriformes	**02**	**LC**	MG736541-542
79	*Akysis manipurensis*	Akysidae	Siluriformes	**02**	**DD**	KX886801, MG736543
80	*Gagata cenia*	Sisoridae	Siluriformes	**03**	**LC**	MG736544-546
81	*Erethistoides sicula*	Sisoridae	Siluriformes	**01**	**DD**	KU667367
82	*Pseudolaguvia virgulata*	Sisoridae	Siluriformes	**01**	**DD**	MG736547
83	*Glyptothorax ngapang*	Sisoridae	Siluriformes	**02**	**LC**	MG736548-549
84	*Glyptothorax manipurensis*	Sisoridae	Siluriformes	**01**	**VU**	MG736550

85	*Glyptothorax telchitta*	Sisoridae	Siluriformes	**01**	**LC**	KU667402
86	*Glyptothorax trilineatus*	Sisoridae	Siluriformes	**02**	**LC**	KU667332-333
87	*Glyptothorax ventrolineatus*	Sisoridae	Siluriformes	**03**	**LC**	MG736551-553
88	*Glyptothorax* sp._01	Sisoridae	Siluriformes	**01**	**-**	MG736554
89	*Clarius batrachus*	Clariidae	Siluriformes	**02**	**LC**	MG736555-556
90	*Heteropneustes fossilis*	Clariidae	Siluriformes	**04**	**LC**	MG736557-560
91	*Macrognathus aral*	Mastacembelidae	Synbranchiformes	**01**	**LC**	KJ936679
92	*Macrognathus puncalus*	Mastacembelidae	Synbranchiformes	**01**	**LC**	KJ936684
93	*Mastacembelus armatus*	Mastacembelidae	Synbranchiformes	**04**	**LC**	MG736561-564
94	*Pillaia indica*	Chaudhuriidae	Synbranchiformes	**04**	**EN**	KJ936644-647
95	*Parambassis ranga*	Ambassidae	Perciformes	**07**	**LC**	KJ936704-708, MG736565-566
96	*Parambassis waikhomi*	Ambassidae	Perciformes	**02**	**NE**	MG736567-568
97	*Nandus nandus*	Nandidae	Perciformes	**04**	**LC**	MG736569-572
98	*Badis assamensis*	Badidae	Perciformes	**04**	**DD**	KU667392, MG736573-575
99	*Badis badis*	Badidae	Perciformes	**02**	**LC**	MG736576-577
100	*Badis ferrarisi*	Badidae	Perciformes	**01**	**LC**	KX886804
101	*Badis tuivaei*	Badidae	Perciformes	**03**	**EN**	KJ936889, MG736578-579
102	*Badis* sp._01	Badidae	Perciformes	**01**	**-**	MG736580
103	*Glossogobius giuris*	Gobiidae	Perciformes	**02**	**LC**	KJ936820-21
104	*Trichogaster lalius*	Belontidae	Perciformes	**03**	**LC**	KJ936699-701
105	*Channa marulius*	Channidae	Perciformes	**08**	**LC**	KJ936892-895, MG736581-584
106	*Channa orientalis*	Channidae	Perciformes	**10**	**NE**	KJ936639-641, KU667382, MG736585-590
107	*Channa punctata*	Channidae	Perciformes	**02**	**LC**	MG736591-592
108	*Channa stewartii*	Channidae	Perciformes	**03**	**LC**	MG736593-595
109	*Channa striata*	Channidae	Perciformes	**06**	**LC**	KJ936897-898,MG736596-599
	**109**	**22**	**7**	**363**		

Supplementary Table 2: Details of sampling location, geological coordinates (longitude & longitude), River/Drainage System and name of the Indian states of Indo-Myanmar Biodiversity Hotspot

**Sl. No.**	**Sampling Location**	**Latitude**	**Longitude**	**River/Drainage system**	**Indian State**
1	Garobada	25º34’37” N	90º01’29” E	Kalu/Brahmaputra	Meghalaya
2	Lalmati	25º45’51” N	89º57’16” E	Jinjiram/Brahmaputra	Meghalaya
3	Siyang,	25°15'53"N	91°58'49"E	Umngot/Brahmaputra	Meghalaya
4	Nongpoh	25°54'30"N	91°52'50"E	Umgthum/Brahmaputra	Meghalaya
5	Bhagma	25°11'42"N	91°22'20"E	Ballat/Brahmaputra	Meghalaya
6	Wilmgaring	25°16'38"N	91°34'26"E	Ballat/Brahmaputra	Meghalaya
7	Sonapur	25°06'09"N	92°21'39"E	Lukha/Brahmaputra	Meghalaya
8	Nongbarch	25°13'59"N	92°01'53"E	Amlayee/Brahmaputra	Meghalaya
9	Walikaba	25°43'48"N	91°40'93"E	Laitryngew/ Brahmaputra	Meghalaya
10	Dapchhuah	23°46'30"N	92°31'16"E	Tut/Barak	Mizoram

11	Reiekkai Tlawng bridge	23°42'31"N	92°40'28"E	Serlui/Barak	Mizoram
12	Tuirial Village	23°43'07"N	92°47'58"E	Tuirial/Barak	Mizoram
13	Arbai Durtlang Village	23°47’43" N	92°45’25"E	Tuivawl/Barak	Mizoram
14	Sihhmui	23°48'11"N	92°37'42"E	Tlawng/ Barak	Mizoram
15	Sairang	23°48’51”N	92°39’06”E	Tlawng/Barak	Mizoram
16	Lengte Kai	23°48’04”N	92°37’34”E	Tlawng/Barak	Mizoram
17	Eden Li	23°31'39"N	92°38'13"E	Tlawng/Barak	Mizoram
18	Champai	23°29’29”N	93°20’46”E	Tuivai/Barak	Mizoram
19	Tlabung	22°54’31”N	92°27’54”E	Khawthlangtuipui/ Karnaphuli	Mizoram
20	Lorphuw	24º19’07” N	94º07’38” E	Chakpi/ Chindwin	Manipur
21	Nunglitpi	24 º11’34”N	94 º10’10”E	Lokchao/Chindwin	Manipur
22	Yuzang	24 º08’01”N	94 º10’22”E	Khujai/Chindwin	Manipur
23	Kumbi	24 º26’01”N	93 º47’52”E	Khuga/Chindwin	Manipur
24	Near Singngat	24°14’53”N	93°54’91”E	Tuivai/Barak	Manipur
25	Modi Village	24°17’84”N	93°58’43”E	Chakpi/ Chindwin	Manipur
26	Hanatham	24°30’58”N	93°97’47”E	Chakpi/ Chindwin	Manipur
27	Tengnoupal	24°19’44”N	94°13’90”E	Lokchao/Chindwin	Manipur
28	Moreh Village	24°24’92”N	94°30’00”E	Lokchao/Chindwin	Manipur
29	Barma Camp	25 º55’32”N	93°44'49"E	Dhansiri/Barak	Nagaland
30	Model village	25 º51’27”N	93 º46’41”E	Chathe/Barak	Nagaland
31	Tchulhumro	26 º02’00”N	94 º13’26”E	Tchulhumro/Barak	Nagaland
32	WalFord Colony	25 º54’26”N	93 º44’41”E	Dhansiri/Barak	Nagaland
33	Mungya Village	26º06’37”N	94º23’14”E	Doyang/Barak	Nagaland
34	Phek	25º50’08”N	94º31’23”E	Tizu/Chindwin	Nagaland
35	Meluri	25º38’34”N	25º35’71”E	Tizu/Chindwin	Nagaland
36	Longsa Village	26º09’18”N	94º20’00”E	Dhiku/Barak	Nagaland
37	Full Nagarjan	25º53’10”N	93º44’09”E	Dhansiri/Barak	Nagaland
38	Aghunato Tizu Bridge	26º02’18”N	94º34’31”E	Tizu/Chindwin	Nagaland
39	Tacholeru	25º36’28”N	94º25’57”E	Tuphaleri	Nagaland
40	Losataphe	25º35’42”N	94º25’78”E	Tuphaleri	Nagaland

Figure Legend

Supplementary Figure 1: NJ analyses (based on K2P genetic distance) of 363 COI barcodes representing 109 species. The phylogenetic tree is drawn to scale (represented in part A and B) and genetic distances were computed using the Kimura 2-parameter method and are in the units of the number of base substitutions per site. The value of the nodes represents the number of percent bootstrap replication.

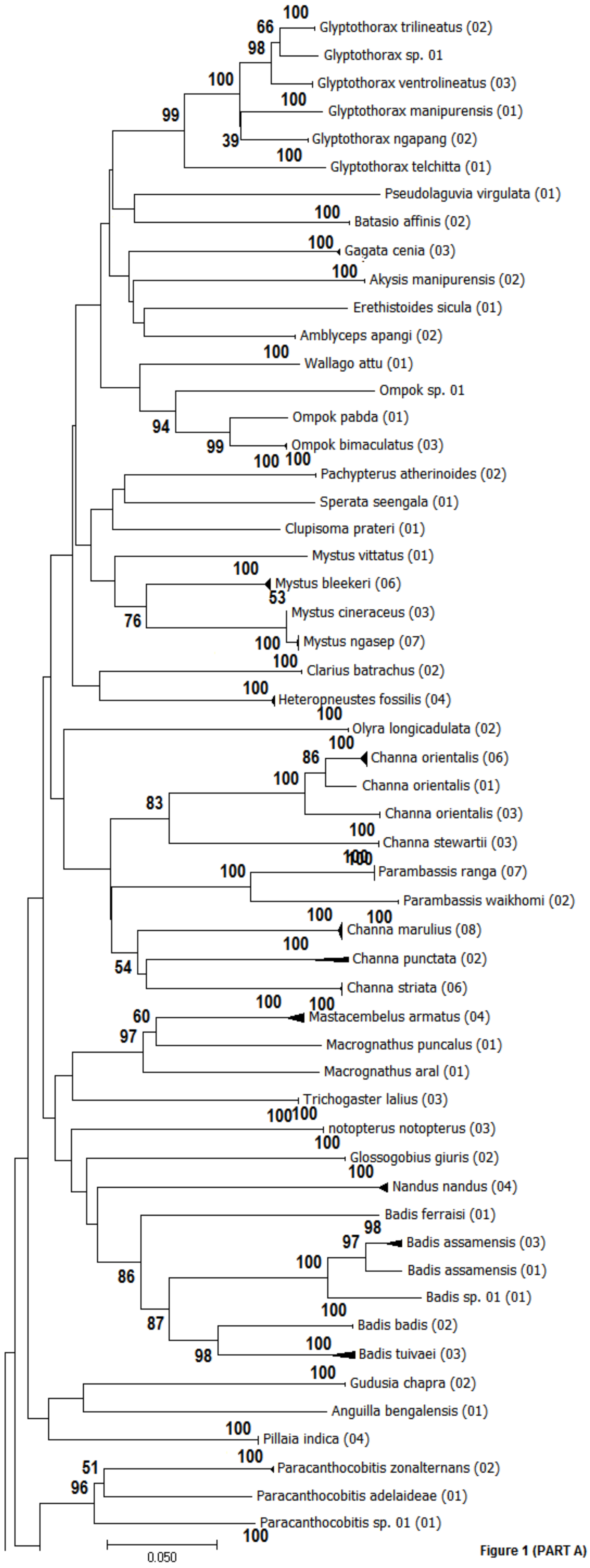

Figure 1 (PART A)

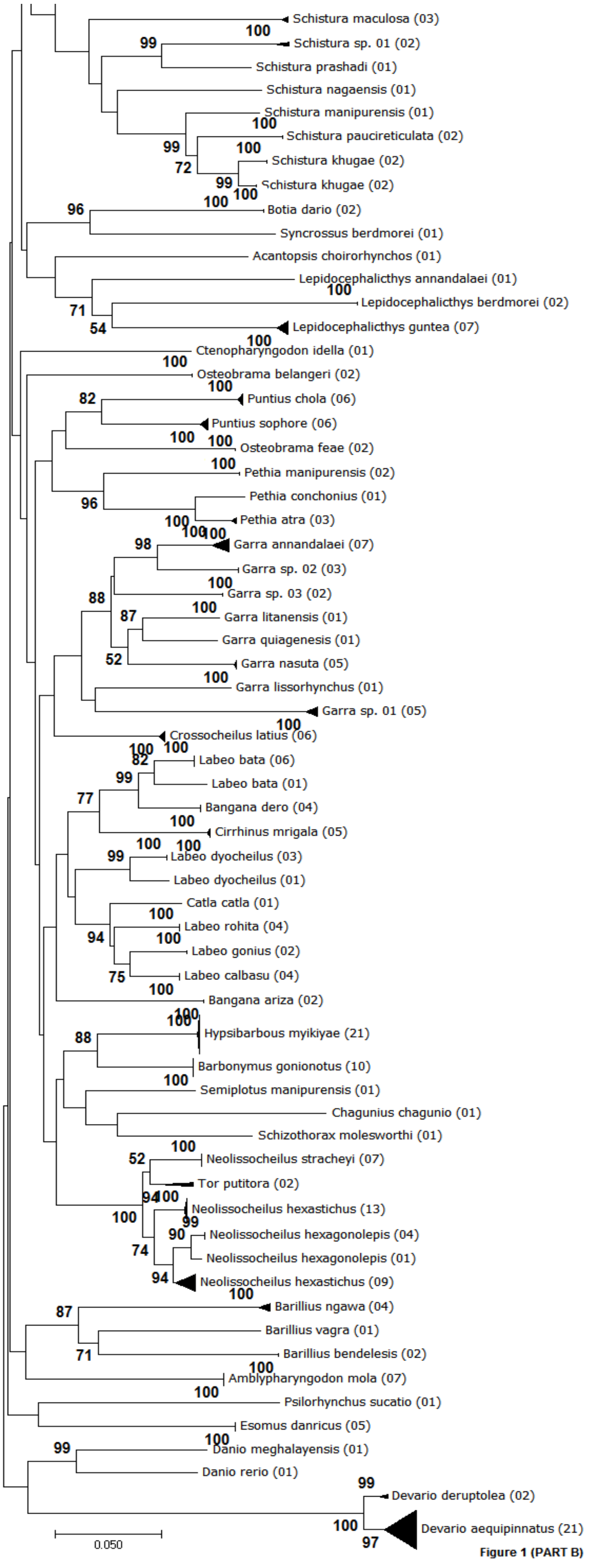

Figure 1 (PART B)
